# Characterization of sensitivity of optical fiber cables to acoustic vibrations

**DOI:** 10.1038/s41598-023-34097-9

**Published:** 2023-05-01

**Authors:** Petr Dejdar, Ondrej Mokry, Martin Cizek, Pavel Rajmic, Petr Munster, Jiri Schimmel, Lenka Pravdova, Tomas Horvath, Ondrej Cip

**Affiliations:** 1 Department of Telecommunications, Brno University of Technology, FEEC, Technicka 12, 616 00 Brno, Czech Republic; 2 Institute of Scientific Instruments of the Czech Academy of Sciences (ISI), Královopolská 147, 612 64 Brno, Czech Republic

**Keywords:** Optical sensors, Imaging and sensing

## Abstract

Fiber optic infrastructure is essential in the transmission of data of all kinds, both for the long haul and shorter distances in cities. Optical fibers are also preferred for data infrastructures inside buildings, especially in highly secured organizations and government facilities. This paper focuses on a reference measurement and analysis of optical fiber cables sensitivity to acoustic waves. Measurement was carried out in an anechoic chamber to ensure stable conditions of acoustic pressure in the range from 20 Hz to 20 kHz. The frequency response, the signal-to-noise ratio per frequency, and the Speech Transmission Index are evaluated for various types of optical fiber cables and different ceiling tiles, followed by their comparison. The influence of the means of fixing the cable is also studied. The results prove that optical fiber-based infrastructure in buildings can be exploited as a sensitive microphone.

## Introduction

Nowadays, optical fibers are increasingly often used for both data and non-data transmission. Many research groups focus on protection of fiber based infrastructures against data eavesdropping that can be done by several techniques^1^. Some data transmissions are not encrypted and even if they are, there is a high risk that in near future, these data will be decryptable by quantum computers. Therefore, the hot topics today are quantum encryption and post-quantum encryption. A relatively unexplored area is fiber optic sensing for vibrations in the acoustic, thus, audible spectrum.

Mechanical vibrations and acoustic noise acting on the optical fiber cause changes in the strain and the refractive index of the fiber core. These changes can subsequently be detected by several methods and converted into an electrical signal followed by acoustic reproduction. Information such as the audio component of a video call, a conversation between people in a room or a phone call can be intercepted even before it is converted into digital form and encrypted. Thus, optical fiber infrastructures, mainly inside buildings, can be used as sensitive microphones, posing a significant security risk. The roots of fiber optic acoustic sensing date back to the 1970s, when the first audible sound sensing experiments were realized^2–4^. Acoustic sensing has recently been a highly studied area^5–7^ because of the security of fiber optic based information systems and networks. Acoustic sensing techniques can be divided on the basis of the methods used.

Fiber strain changes can be detected in Rayleigh backscattering. The distributed acoustic sensing technique (DAS) uses this effect, where a coherent laser pulse is transmitted along an optical fiber^8^. The scattering spots in the fiber cause the fiber to act as a distributed interferometer. The intensity of the reflected light is measured as a function of time after transmitting the laser pulse. DAS detects pico-strain-level signatures in the fiber induced by vibroacoustic disturbances caused by an event near the optical cable. These perturbations change the scattering in the fiber core at a molecular scale, originating from the sub-wavelength heterogeneities formed when the fiber is drawn. Further research is focused on the Phase-sensitive Optical Time-Domain Reflectometry ($\Phi$-OTDR) technology^9^.

Changes in the refractive index of the fiber core caused by external mechanical vibrations and acoustic noise lead to Doppler shifts of light waves travelling through an optical fiber. This phenomenon can be explained as a Doppler effect in a flexible and expandable waveguide^10^. Doppler-induced frequency or phase shift of a propagating light wave is detectable in schemes of optical interferometers where the instantaneous interference phase in the time domain is converted to the electric signal^11^. The frequency shift is detectable in an arrangement Fabry–Perot (FPI), Mach–Zehnder (MZI) or Michelson (MI) interferometers formed by optical fibers with necessary optical elements included in the optical setup.

The FPI is very often used for the arrangement of point-optical microphones. Variety of FPI based microphone designs are available^12–16^ and dependences of the cavity length and the materials used can be compared. Such microphones can also be used for multipoint sensing, for example, using a 1:4 splitter^17^.

A special use of the FPI is possible^18^ where the multimode–singlemode–multimode (MSM) structure and direct measurement detection are used to detect acoustic vibrations. Fiber Bragg grating (FBG) microstructures^19^ incorporated in the sensing optical fiber can be used as mirrors for the FPI where an optical cavity is formed between two or more FBGs. The FPI arrangement is suitable for the use as microphones and hydrophones as well^20,21^. Several works based on the FPI arrangement have been devoted to voice sensing with ethylene propylene diene terpolymer film and the aluminium surface^17^ and based on cellulose triacetate diaphragm^13^. There are also unique variants of the detection schemes in the arrangement with the FPI. They include an experiment using a laser feedback interferometer, where changes of the refractive index of the sensing fiber lead to changes of optical frequency of the detecting laser^22^. An important disadvantage of the FPI-based techniques for acoustic sensing is the limited possibility of measuring at one only or very low number of points on the optical fiber. The other disadvantage is the need for a specially modified fiber, e.g. with FBG microstructures.

Arrangements using the MZI for acoustic sensing are used for example, it is possible to use microfiber MZI^23^ which again requires a special fiber, or to use conventional fibers for acoustic monitoring of gas turbines^24^. It is also possible to use the open cavity and the collimators in the sensing arm of the MZI for sound sensing^25^.

Arrangements of the MI are often used as hydrophones sensing ultrasound^26^ but also as sensors for audible frequencies^27^. Implementations in sensing seismic vibration have been also reported^28^ as well as possible use in monitoring of marine structures^29^. It is also worth noting that research is carried out that deals with improving the noise stability of the MI^30^. The star topology of the fiber optic infrastructure inside buildings gives the opportunity to build the MI arrangement. A single optical fiber usually runs from the room with the central optical switch to the room with a piece of terminal equipment. The fiber can thus sense acoustic signals along its entire route and can be connected as a measuring arm of an MI arrangement.

In this paper, we set up an experimental MI that allows the detection of acoustic signals through an optical fiber guided by different types of corridors. We focused on measuring the sensitivity of this arrangement to defined acoustic signals in a fully anechoic laboratory. The experiments examined the influence of several factors such as the optical fiber position and types of optical fibers on the quality of the detected signal regarding a level of speech intelligibility. The properties of the acquired signals were analyzed, the individual measurements’ frequency responses were compared, and signal-to-noise ratios were investigated. In our work, we also measure and evaluate the Speech Transmission Index (STI), which is the prevailing way to objectively assess the expected intelligibility of speech signals after passing through a system.

## Methods

**Figure 1:**
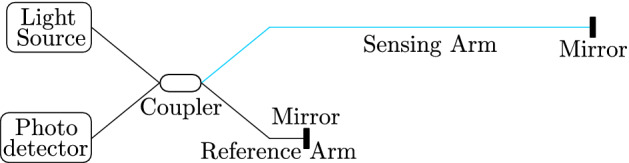
Schematic diagram of the MI^33^.

The Michelson interferometer is widely utilized for its flexibility of use. A fiber arrangement contains only a single coupler through which coherent light is distributed from the laser source to the sensing and reference arm, as seen in Fig. 1. The output light intensity can be calculated, in a simplified version, assuming a 50:50 splitter and no attenuation, according to the formula^31^

1 $$\begin{aligned} I=I_o \cos ^{2}\left[ \frac{\pi }{\lambda }\left( n_s L_s -n_r L_r \right) \right] \end{aligned}$$

where $I_{o}$ is the constant intensity of output light from splitter, $L_s$ and $n_s$ are the length and refractive index of the sensing fiber, respectively, $L_r$ and $n_r$ are the length and refractive index of the reference fiber, respectively. Finally, $\lambda$ is the wavelength. At the end of these arms, there are mirrors or Faraday mirrors reflecting light to the coupler, which again merges the beams, and the photodetector detects the light. Since the MI reflects all the power back to the coupler, it is advisable to use an isolator that prevents damage to the laser. As the light passes through the arms twice, the optical phase shift per length is doubled. Thus, the MI is more sensitive than the MZI^32^.

For the detection of mechanical and acoustic vibrations, MI is often used, as stated, for example, in the review by Li et al.^34^ Among other issues, the review discusses current methods of demodulation in fiber interferometers. Another article^35^ deals with noise removal using MZI, MI, and $\Phi$-OTDR combined. For the MI, it is also possible to expand the number of arms, which makes it possible to localize the sound source^36^.

**Figure 2:**
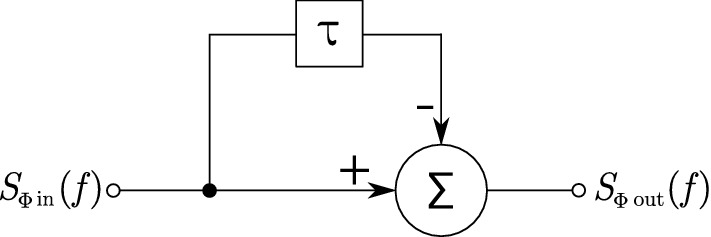
Signal-flow graph of the self-heterodyne effect observed in the MI. $S_{\Phi \,in }(f)$ the power spectral density of laser phase noise at the input, $S_{\Phi \,out }(f)$ the power spectral density of the interference phase at the output, $\tau$ the total difference of time of flight between the two arms of the interferometer.

Due to the self-heterodyne effect, the phase noise of the laser needs to be taken into account in both MI and MZI^37,38^. The phase fluctuations of the laser can be observed as additive noise in the detected interference phase. Considering the phase noise of the laser as the input signal and the noise in the detected interference phase as the output signal, the interferometer acts as a feed-forward high-pass comb filter as shown in Fig. 2.

The transfer function of such a system can be expressed as

2 $$\begin{aligned} H_{\Phi }(s) = \frac{\Phi _{out }(s)}{\Phi _{in }(s)} = \frac{\Phi _{in }(s) - \Phi _{in }(s)\textrm{e}^{-s\tau }}{\Phi _{in }(s)} = 1 - \textrm{e}^{-s\tau } . \end{aligned}$$

**Figure 3:**
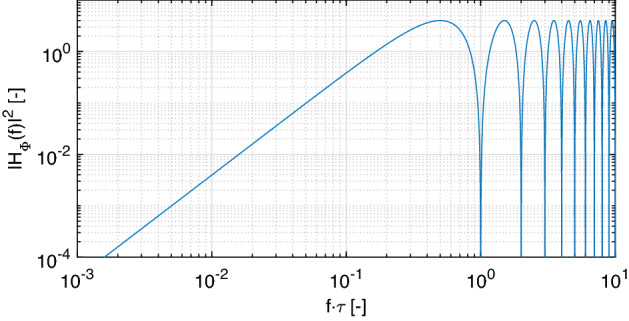
Transfer function $\vert H_{\Phi }(f)\vert ^2$ for the laser phase noise observed in the detected interference phase. The frequency axis is normalized to $1/\tau$.

After substituting $\textrm{j}2\pi f$ in the place of *s* the magnitude of the transfer function can be expressed:

3 $$\begin{aligned} \vert H_{\Phi }(f) \vert ^2 = 4\sin ^2(\pi f \tau ), \end{aligned}$$

see Fig. 3. The power spectral density of the output interference phase noise then relates to the input laser phase noise power spectral density as follows:

4 $$\begin{aligned} S_{\Phi \,out }(f) = \vert H_{\Phi }(f)\vert ^2~S_{\Phi \,in }(f). \end{aligned}$$

Practically, this means that the longer the length difference $\Delta L$ between the sensing and reference arms and the broader the desired frequency range, the more coherent laser source is required for the application. For example, a MI with $\Delta L = 100$ m has $1/\tau = 1$ MHz and the power of the laser phase noise gets attenuated by 24 dB at 10 kHz. In our experiments we use a RIO ORION module with a linewidth of 1 kHz, which is in practice suitable well for audible frequency sensing applications with $\Delta L$ up to the kilometer range.

A characterization of optical fibers and cables as acoustic sensors mainly for speech is probably of the greatest interest in real infrastructures, for example for the sake of security. Despite this fact, our experiment consists of sensing acoustic vibrations in a maximally controlled environment. Our measurements are thus performed in an anechoic room. This allows us to separate inherent factors from those affecting the quality of the signal in the real case. As far as we know, only a single similar experiment was carried out, Zhang et al.^39^ describing the calibration of the optical fiber sensor; it contains only basic measurements, neither measurements of human speech nor a comparison of intelligibility to reference microphones.

## Experiments

### Measurement setup

**Figure 4:**
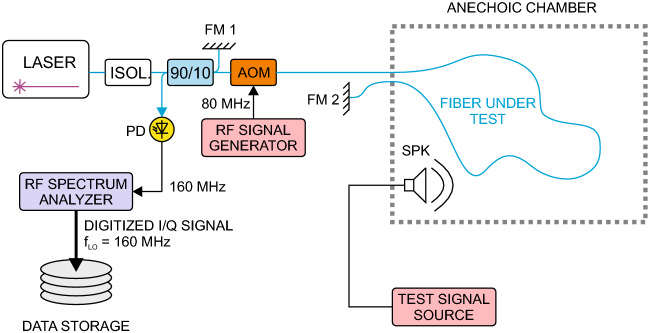
Schematic diagram of the heterodyne Michelson interferometer setup. *AOM* acousto-optic modulator, *FM* Farraday mirror, *ISOL* optical isolator, *PD* photodetector, *LASER* RIO ORION @ 1540 nm, *SPK* speaker box, *90/10* fiber coupler. RF signal generator and RF spectrum analyzer are slaved to a common 10MHz reference. The RF amplifiers and filters are omitted for clarity. In certain measurements, FM2 was placed into the anechoic chamber alternatively.

For measuring the transmission of acoustic vibrations to the fiber we have set up a heterodyne Michelson interferometer (MI) configuration shown in Fig. 4. The sensing arm of the interferometer was formed of the optical fiber under test leading through the controlled environment of the anechoic chamber where it is exposed to acoustic vibrations generated by a loudspeaker system.

#### Heterodyne fiber optic interferometer

The heterodyne MI setup used is an extension to a classical homodyne MI shown in Fig. 1. Instead of detecting the interference phase as a DC intensity level at the photodetector, the interference phase is observed as a phase shift of the detected radiofrequency (RF) beat note. The heterodyne MI setup uses an acousto-optic modulator (AOM) to shift the optical frequency between the reference arm and sensing arm by a specific amount. In our case it is 2 $\times$ 80 MHz since the light passes the AOM twice. The light waves returning from both the arms of the MI are non-linearly mixed at the photodetector, producing a RF beat note. The central frequency of the beat note is equal to the total frequency shift between the MI arms, i.e. 160 MHz in our case. Exposition of the sensing arm to acoustic vibrations results in slight changes of its optical length. These can be observed as a phase modulation of the detected RF beat note, or in other words, as its phase shift from a reference 160 MHz local oscillator signal. Since in the heterodyne detection technique, only the detected phase is relevant the method is immune to intensity fluctuations of the optical signal.

To allow for a greater versatility in signal processing, the demodulation of the beatnote signal is carried out off-line on sets of digitally recorded RF signal samples. As illustrated in Fig. 4, the RF signal is converted down using a real-time RF spectrum analyzer (Signal Hound USB-SA44B) in the zero-span regime to the baseband in-loop and quadrature (I/Q) signal components, which are digitized and stored. The I/Q signal components contain the complete information about the RF signal magnitude and instantaneous phase relative to the local oscillator (LO) of the spectrum analyzer with frequency $f_{{LO} }$ = 160 MHz. The sampling rate of the baseband signal is 486 kS/s, which provides approximately 20-fold oversampling of audible frequency band signals. Equations (5a) and (5b) describe the relationship between the instantaneous phase samples $\phi _n$ in time (relative to the reference LO) and magnitude samples $M_n$ of the RF signal and I/Q component samples $I_n$ and $Q_n$:

5a $$\begin{aligned} \phi _n&= {\text {atan2}}\left( Q_n, I_n\right) ,\; \phi _n \in ( \,-\pi ,\pi \rangle , \end{aligned}$$

5b $$\begin{aligned} M_n&= \sqrt{I_n^2 + Q_n^2}, \end{aligned}$$

where ${\text {atan2}}$ denotes the four-quadrant inverse tangent.

To get the interference phase $\Phi$ whose change is directly proportional to optical path length change, we need to unwrap the instantaneous phase $\phi$ of the RF signal by the algorithm described for example in the Matlab documentation^40^. Doing so extends the interval of possible phase angle values seamlessly from $(\,-\pi ,\pi \rangle$ to $(-\infty ,\infty )$. The resulting samples of $\Phi$ are used as the primary input audio signal for analyses discussed in the following sections of the manuscript.

#### Anechoic chamber

**Figure 5:**
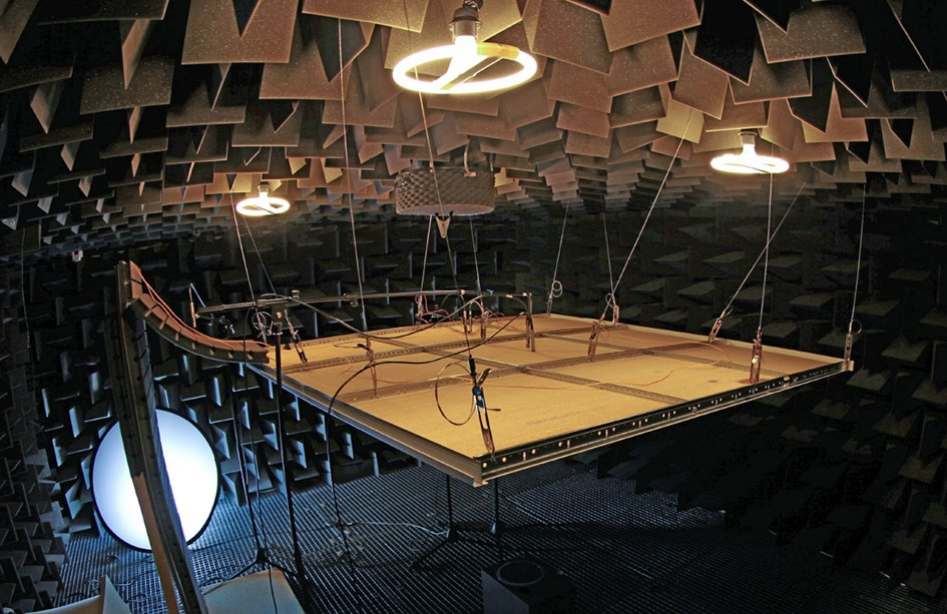
Photo of the setup in the anechoic chamber.

The anechoic chamber utilized for the measurements has a volume of 90 m$^3$ and its critical frequency is about 120 Hz. A simulated ceiling structure for carrying 3$\times$3 ceiling panels of standard size 60$\times$60 cm was installed in the center of the chamber. A two-band Event 20/20 loudspeaker system was used as the sound source. The reference axis of the loudspeaker system was oriented perpendicularly to the structure carrying the fiber under test in its center point. The compensation of sound pressure dependence on frequency due to loudspeaker system frequency response and room modes at low frequencies was done using the B & K Type 4190 measurement microphone mounted at the structure as a boundary microphone in the reference axis of the loudspeaker, see Fig. 12. The structure and the loudspeaker system were installed using mechanical damping elements to minimize transmission of vibrations into the fiber under test by other means than the acoustic waves, see Fig. 5. The Audio Precision APx525 acoustic analyzer was used for the measurement, with one channel driven by the compensation microphone and the other by the audio signal detected by the interferometer. All the measurements were done in closed loop with analyzer in sync with generator.

In the given setup, we aim at the following comparisons:

1. the effect of the panel type,
2. the effect of the cable/fiber type,
3. the effect of the cable/fiber position with respect to the ceilings panels,
4. the effect of the FM2 mirror position.

### Test signals

To measure and both visually and numerically evaluate the sensing capabilities in different setups, we performed three types of measurements, resulting in the following quantities:

1. frequency response of the system using a swept sine excitation signal,
2. signal-to-noise ratio per frequency (SNRf) using a stepped sine excitation signal,
3. the Speech Transmission Index for Public Address systems (STIPA).

Measurement of the frequency responses using the swept sine signal, as well as the estimation of speech intelligibility using the STIPA protocol are common techniques in signal processing and system analysis, see the description below. The metrics are measured on audio signals obtained from the acquired I/Q signals using phase demodulation. The signals are downsampled to $F_s = 48$ kHz.

### Signal processing

We treat all the (already discrete, finite-length) signals as column vectors, i.e. a signal $\textbf{x}$ of length *N* is denoted $\textbf{x}= [x_1, x_2,\dots ,x_N]^\top$. We index matrices in a similar manner, i.e. matrix $\textbf{A}$ of size $M\times N$ contains elements $a_{i,j}$ for $i=1,\dots ,M$ and $j=1,\dots ,N$.

#### Frequency responses

**Figure 6:**
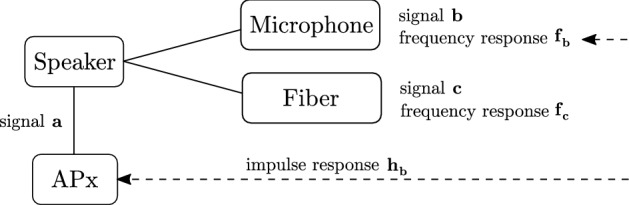
Schematic of the frequency response estimation.

One of the most common means to characterize properties of a linear time-invariant system *S* is using its impulse response $\textbf{h}_S$ or its frequency response $\textbf{f}_S$, related to the former through the Fourier transform $\mathscr {F}$ by a standard formula^41^ $\textbf{f}_S = \mathscr {F}(\textbf{h}_S)$. The frequency response is commonly estimated using a linearly or exponentially swept sine signal $\textbf{a}$, which, as the input signal, is transformed by system *S* to produce an output $\textbf{b} = S(\textbf{a})$. The response is then calculated^42–44^ as $f_S = \frac{\mathscr {F}(\textbf{b})}{\mathscr {F}(\textbf{a})}$. We estimate the response using the acquired microphone signal (denoted $\textbf{b}$) and the corresponding impulse response ($h_{\textbf{b}}$) as obtained from the APx, see Fig. 6. Denoting the (unknown) source signal $\textbf{a}$, it holds $h_{\textbf{b}} = \mathscr {F}^{-1}(\frac{\mathscr {F}(\textbf{b})}{\mathscr {F}(\textbf{a})})$. The desired frequency response is

6 $$\begin{aligned} \textbf{f}_{\textbf{c}} = \frac{\mathscr {F}(\textbf{c})}{\mathscr {F}(\textbf{a})} = \frac{\mathscr {F}(\textbf{c})}{\mathscr {F}(\textbf{b})} \cdot \frac{\mathscr {F}(\textbf{b})}{\mathscr {F}(\textbf{a})} = \frac{\mathscr {F}(\textbf{c})}{\mathscr {F}(\textbf{b})} \cdot \mathscr {F}(\textbf{h}_{\textbf{b}}), \end{aligned}$$

where both the signals $\textbf{b}$ (from the microphone) and $\textbf{c}$ (demodulated from the fiber) and the response $\textbf{h}_{\textbf{b}}$ (provided by the APx) are available. The division and multiplication in Eq. (6) are performed elementwise.

#### Step frequency analysis

The second input test signal consists of 50 subsequent time segments, each of them is occupied by a single, pure sinusoid of a prescribed frequency *f*. In our experiments, we used 53 values of the target frequency *f* logarithmically spread from 50 Hz up to 20 kHz. To analyze the signal from acquired at the fiber, we first divide the acquired signal into segments, corresponding to the input signal as described above. Then, each segment $\textbf{x}$ is decomposed into the useful signal $\hat{\textbf{x}}$ (sinusoid with given frequency *f*) and noise $\textbf{n}$, such that $\textbf{x}= \hat{\textbf{x}} + \textbf{n}$ and $\textbf{n}$ is minimal in the least square sense. It is then straightforward to compute the SNR corresponding to the source frequency *f*.

In more detail, we search for the signal $\hat{\textbf{x}}$ (note that by choosing the parameters *a*, *b*, such a sum generates a sinusoid with fixed frequency *f* and arbitrary amplitude and phase)

7 $$\begin{aligned} \hat{x}_m = a\cdot \cos (2\pi f (m-1)/F_s ) + b\cdot \sin (2\pi f (m-1)/F_s ), \end{aligned}$$

where $m=1,\ldots ,M$. Real numbers *a*, *b* are parameters to be optimized such that the energy of the noise $\textbf{x}- \hat{\textbf{x}}$ is minimized. The length *M* is the length of a single step in the sequence, in our case $M = 1.6\cdot F_s = 76\,800$ samples for step length set to 1.6 s. The described task is a linear regression problem. Using the design matrix $\textbf{X}$ of size $M\times 2$, $x_{m,1} = \cos (2\pi f m/F_s )$, $x_{m,2} = \sin (2\pi f m/F_s )$, the optimal parameters and the denoised signal can be found explicitly^45^ as

8 $$\begin{aligned} \begin{bmatrix} \hat{a} \\ \hat{b} \end{bmatrix} = \left( \textbf{X}^\top \textbf{X}\right) ^{-1}\textbf{X}^\top \textbf{x}, \quad \hat{\textbf{x}} = \textbf{X} \begin{bmatrix} \hat{a} \\ \hat{b} \end{bmatrix}. \end{aligned}$$

Then, we may easily compute SNRf for the specific frequency *f* as

9 $$\begin{aligned} SNRf (f) = 10\cdot \textrm{log}_{10}\frac{\sum _{m=1}^{M} \vert \hat{x}_m \vert ^2}{\sum _{m=1}^{M} \vert x_m - \hat{x}_m \vert ^2}, \end{aligned}$$

where the SNRf value is expressed in decibels.

#### Speech Transmission Index for Public Address Systems

**Figure 7:**
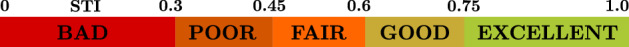
STI scale and the corresponding speech intelligibility.

STIPA is an established method for objective assessment of speech intelligibility after passing the speech signal through a system^46^. The standardized test signal contains pink noise, amplitude-modulated in seven non-overlapping bands. The bands, frequencies and modulation depths are prescribed such that the resulting noise signal is statistically similar to male speech. At the output, the signal is analyzed in terms of loss of amplitude and modulation depth, and a final number (STI, Speech Transmission Index) in the interval from 0 to 1 is computed. See Fig. 7 for the reference values.

### Results

First, we present the comparison for particular choices of the panel:

- no panel (none),
- standard panel (standard),
- AMF ECOMIN Filigran acoustic panel (acoustic).

**Figure 8:**
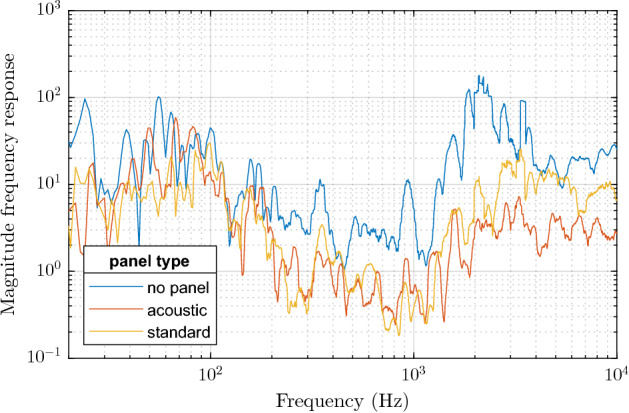
Smoothed magnitude frequency characteristics for different panel types. For frequencies above ca. 2 kHz, the decreasing response of the channel gets gradually overwhelmed by the self-heterodyne phase noise of the laser and therefore this frequency range should not be taken into account in the comparison.

**Figure 9:**
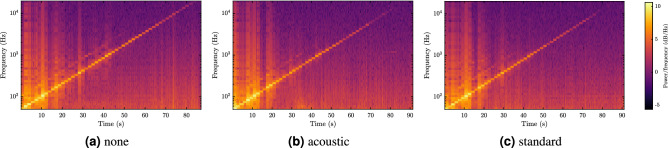
Spectrograms for the step measurement with different panel types. The color scale is the same across the three spectrograms. Note that the frequency bins correspond to the measured frequencies, i.e. they are not equidistant as in the commonly used short-time Fourier transform.

**Figure 10:**
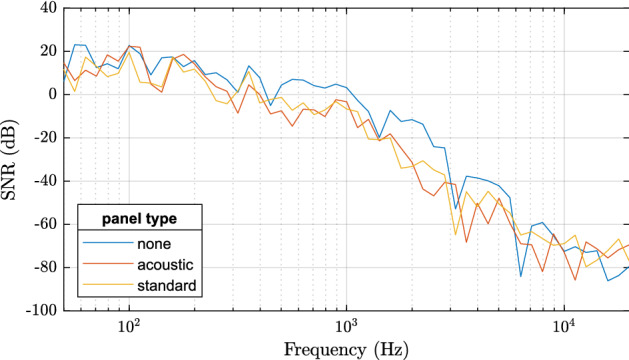
SNRf for different panel types.

The results are presented in terms of the frequency response in Fig. 8 and in terms of the step analysis in Figs. 9 and 10, where the standard Patch Cord G.657.A1 (PC) hanging above the panel was used for the measurement. Note that the frequency response is very noisy due to the nature of the measurement, especially for high frequencies. Therefore, we used fractional-octave smoothing^47^ for the visualizations, using of 1/12th-octave.

As expected, we observe in Fig. 8 that the response with no panel is stronger than with either the acoustic or standard panel. The difference between the two panel types is visible only in parts of the spectrum, especially for very low frequencies. However, we also observe that the frequency response is very noisy and even degraded for frequencies above ca. 1 kHz. In that range, the response is very strong, however, the signal consists of mostly noise, making the result unreliable. This motivates the SNR analysis of the stepped frequency measurements—Fig. 9 shows the measured signals in the time-frequency domain, while Fig. 10 presents the SNRf values.

**Figure 11:**
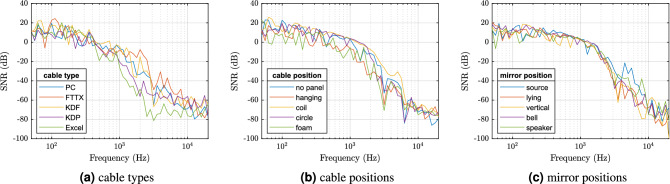
Comparison of different setups in terms of SNRf.

We observe in Fig. 9 that both panel types partly work as a low-pass filter—see top right corner of the spectrograms, where the magnitude of the signal (visible as the diagonal of the temporal spectrum) decreases in both cases (Fig. 9c,d). Furthermore, the whole system introduces a significant level of noise, which depends on the input signal frequency.

Finally, we focus on SNRf in Fig. 10. Note that the values are globally very low. This is caused by the experiment setup where the useful signal is extremely narrow in the frequency domain, since it consists of only a single frequency. On the other hand, the noise energy corresponds to the whole remainder of the frequency range. Thus, we focus only on the relative, not absolute comparison of the results. Figure 10 demonstrates that the level of noise with respect to the signal is lower when no panel is present (i.e. the SNR is higher). However, when the panels are present, the dependency on the particular type is as expected (acoustic panel transfers less signal), but it does not appear to be quite significant.

For the comparison of cable type, we tested the following possibilities:

- standard Patch Cord G.657.A1 (PC)—$\oslash$2.0 mm, Yellow PVC, Furcation Tubing, kevlar threads,
- FTTX 12 fibers G.657.A1 (FTTX)—$\oslash$6.0 mm, PE outer jacket, loose tube, gel,
- KDP flat drop 2 fibers G.657.A1 (KDF)—2.0 $\times$ 3.0 mm, FR-LSZH outer jacket, 2$\times$ dielectric strength member $\oslash$0.5 mm,
- KDP 24 fibers G.657.A1 (KDP)—$\oslash$10.1 mm, FR-LSZH outer jacket, Waterblocking E-glass, Waterblocking yarn, Gel filled loose tube with optical fibers,
- Excel LSOH 24 G.652D fibers OS2 (Excel)—$\oslash$8.5 mm, FR-LSZH outer jacket, tight tube, E Glass strength member.

For the analysis, we chose the position of the cable hanging above the panel since this setting eliminates possible inaccuracies in arrangement of different cables. Based on the observation above, we focus on the results of the step analysis in Fig. 11a.

As expected, we see that the cables with a thicker coating (KDP, Excel) exhibit a drop of SNRf on lower frequencies, compared to the thinner options (PC, FTTX, and KDF). On the other hand, the remarkable observation is the relatively high value of SNRf in the case of the FTTX cable around 2 kHz. The increased sensitivity of the FTTX cable compared to others is most likely caused by the material composition of the cable jacket. It is a hard PE plastic that transmits acoustic vibrations to the fibers.

**Figure 12:**
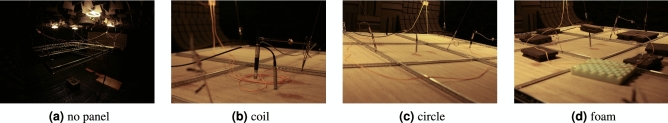
Photos of individual way of placing the FUT.

In terms of the cable position, we have tested several setups, see also Fig. 12:

- hanging above the grid, with no panels present, for reference (no panel),
- hanging above the panel (hanging),
- lying in a coil on the panel (coil),
- lying on the panel in a circle around the perimeter (circle),
- lying on a polyurethane foam on the panel in a circle around the perimeter (foam).

**Figure 13:**
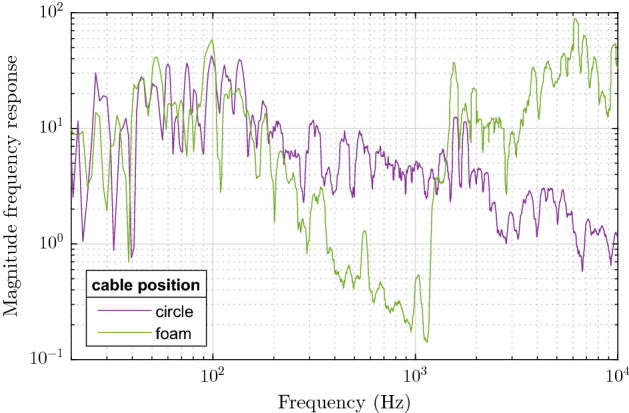
Smoothed magnitude frequency characteristics depending on whether the FUT lies on a foam layer or not.

Clearly, the foam layer serves as a damping element in the whole system, similar to a low-pass filter. While this is more pronounced in terms of frequency response (which reflects the actual amplitude of the signal, see Fig. 13 between 100 Hz and 1 kHz), we observe it also in Fig. 11b when comparing the foam option with the others except for the case of hanging cable. In this latter case, the values of SNRf are also diminished, which is caused by the fact that the vibrations of the panel are transferred to the fiber under test (FUT) indirectly via another air layer, in contrast to all other cases where the FUT lies directly on the panel.

**Figure 14:**
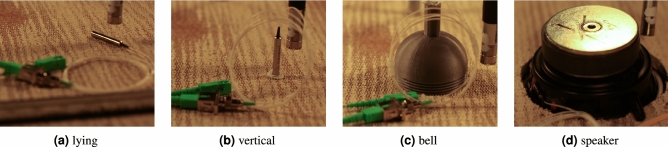
Photos of individual way of placing FM. The FUT is disconnected in the middle in these setups and the FM is placed there.

Finally, we evaluate the effect of the FM2 mirror position, aiming to investigate whether the mirror itself serves as an optical microphone, or an amplifier for the effect of the whole FUT. We test the following setups, see Fig. 14 for illustration of the setups:

- FM located on the side of the laser source (source),
- FM in the middle of the FUT lying on the panel (lying),
- FM in the middle of the FUT vertically glued to the panel (vertical),
- FM in the middle of the FUT in a plastic bell (bell),
- FM in the middle of the FUT glued to a non-playing speaker (speaker).

**Figure 15:**
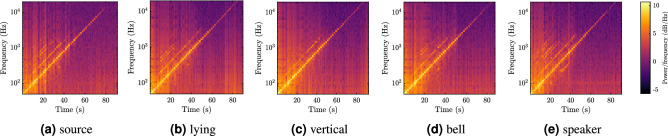
Spectrograms for the step measurement with different mirror positions. The color scale is shared by all the spectrograms.

The results of the step analysis in Fig. 11c do not reveal any clear patterns, suggesting that the integrated effect of the whole FUT outweighs possible inputs to the signal stream caused by the mirror itself. However, there is some distinction observable in the range between 100 and 200 Hz, where the SNR for the source and speaker variants drops significantly. In contrast, the vertical and bell options reach very high values of SNR in this range. The vertical option draws even more attention when visually comparing the spectrograms from the step measurement, see Fig. 15. One may notice that the levels of noise, compared to the clearly observable steps, are mostly similar for all the investigated options (note that the frequency bins are centered on the examined frequencies and the color scales are identical). However, the option including vertically positioned mirror shows (subjectively) the least amount of harmonic distortion among all the options. This can be observed by focusing on the number of visible harmonics (lines parallel to the main diagonal), which is largest for the source and speaker options and smallest for the vertical option. Note that this phenomenon is not numerically evaluated since neither the SNR-based analysis nor STIPA (see the discussion below) revealed any significant effect which could be attributed to the harmonic distortion.

Concerning the speech transmission in terms of STIPA, the values for all the setups are displayed in Table 1. In terms of the panel type, the STI significantly decreases when panels are present and block the direct acoustic waves. This is in correspondence with Fig. 10. The standard panel type surprisingly blocks the signal better than the acoustic panel type. However, one should be careful about strict judgments since the uncertainty of the STI measurement^46^ is 0.02–0.03, and, during the swap of the panels, it could happen that the fiber changed its position or shape slightly.

**Table 1:** Dependence of STI on various setups.

	STI
Panel type	
No panel	0.60
Standard	0.46
Acoustic	0.49
Cable type	
PC	0.46
FTTX	0.66
KDF	0.31
KDP	0.23
Excel	0.2
Cable position	
No panel	0.60
Hanging	0.46
Coil	0.67
Circle	0.67
Foam	0.44
Mirror position	
Source	0.62
Lying	0.65
Vertical	0.54
Bell	0.47
Speaker	0.59

In terms of the cable position, the lowest STI is observed with a hanging cable and with the cable placed on foam blocks. In these cases, the cable is blocked from mechanical vibrations coming from the panel itself. These vibrations are generated by the acoustic waves from the audio source, and play a crucial role in a surprisingly high increase of the STI value. The STI in these two cases is even greater than when panels are removed. The panel plays the role of a resonator here.

The cable type seems to be an imperative factor in the transmission of speech. While the best STI score by far has been achieved with the FTTX cable (0.66), the KDP cable is much more resistant to vibrations, reaching the lowest STI of 0.23. The ranking of the cables corresponds with the frequency-dependent SNRf analysis presented in Fig. 11.

In the setup with a Faraday mirror, its position affects the resulting STI, but this effect is not as pronounced as the effect of the cable type. The best score of STI 0.65 has been achieved with the mirror lying on the panel. Yet, such a high value should be probably attributed to the fact that only in this particular setup, the resonator effect of the panel, as described above, happened. See Fig. 14a where it is evident that the fiber is in firm contact with the panel, in contrast to the other setups.

## Conclusion and future work

In the paper, we focused on the design of a reference method for measuring the sensitivity of optical cables to acoustic vibrations in the audible spectrum. In addition to evaluating the sensitivity of several different fiber optic cables, the influence of the ceiling panel type on the transmission quality was also evaluated. The most sensitive optical fiber sensor systems as well as standard audio microphones were used for the measurements, thanks to which it was possible to obtain relevant information about acoustic wave propagation. In order to consider the measurement as a reference, anechoic chamber/room was used and three different signal processing methods were proposed. The results show that optical cable infrastructures inside buildings can be used as sensitive microphones and can pick up human speech.

In future work, we would like to focus more on the post-processing of the acquired audio signals and, with the use of suitable algorithms, achieve an improvement in the quality of the obtained audio signals. We would also like to focus on measurements in real conditions, i.e. in a room with existing optical infrastructure primarily used for data transmission. In real conditions, degradation of the measurement quality can be expected due to a large number of different sources of interference. However, thanks to the data obtained from the reference measurement in the anechoic chamber, we believe that with appropriate post-processing we will be able to suppress some of the interferences and increase the quality and intelligibility of audio.

## Data Availability

The datasets used and/or analysed during the current study available from the corresponding author on reasonable request.
